# A modified Radiographic Union Score for Tibia (RUST) scoring system for patella fractures treated with osteosynthesis shows excellent intra und interobserver reliability

**DOI:** 10.1007/s00402-024-05736-1

**Published:** 2024-12-27

**Authors:** Julia Elisabeth Lenz, Lorenz Huber, Dominik Szymski, Volker Alt, Markus Rupp, Johannes Weber

**Affiliations:** University Hospital Regensburg, Regensburg, Germany

**Keywords:** RUST, Patella, Fracture, Osteosynthesis, Reliability

## Abstract

**Introduction:**

Patellar fractures are rare at 1% incidence of all fractures. However, they can cause significant functional impairments due to the patella’s role in knee joint extension. Current scoring systems lack objectivity in assessing patellar healing. This study aims to validate the Radiographic Union Score for Tibia (RUST) using biplanar radiographs for assessing surgically treated patellar fractures.

**Materials and methods:**

A retrospective analysis of radiological follow-up examinations was conducted on patients undergoing surgical treatment for patellar fractures from January 1st 2013, to June 30th 2023. Thirty patients were randomly selected, yielding 105 postoperative X-rays representing various healing stages. The modified Radiographic Union Score for Tibia (RUST) was applied to these X-rays by three independent trauma surgeons. Radiological follow-up examinations were randomized, pseudonymized, and stored on a hospital server for blinded assessment by three raters. The modified RUST assessed continuity of patellar borders and cortexes, assigning scores based on cortical bridging. Interobserver and intraobserver reliability were evaluated using intraclass correlation coefficients (ICC), adhering to recommended sample size criteria and interpretation guidelines.

**Results:**

The mean modified RUST was 9.1 ± 2.2 points, with scores ranging from 4 to 12 points. The interobserver intraclass correlation coefficient (ICC) was 0.88 (95% CI, 0.81–0.92) and the intraobserver ICC were 0.6 (95% CI, 0.65–0.84), 0.80 (95% CI, 0.71–0.87) and 0.98 (0.98–0.99) respectively, which indicated good to excellent agreement.

**Conclusions:**

This study validated the Radiographic Union Score for Tibia (RUST) for evaluating bone healing in patellar fractures treated with osteosynthesis, demonstrating good intra- and interobserver reliability. The modified RUST can provide a standardized method for assessing healing in patellar fractures, benefiting both clinical practice and clinical trials.

## Introduction

Patellar fractures are rare fractures, comprising approximately 1% of all fracture occurrences, yet they can result in significant functional impairments due to the critical role of the patella in the knee joint’s extensor mechanism [8, 10]. Particularly in transverse fractures and multifragmentary patellar fractures, open reduction and internal fixation by tension band wiring, screw osteosynthesis, equatorial cerclage, plate osteosynthesis or a combination of techniques plays a crucial role in restoring knee joint function [9, 17]. However, delayed fracture healing with patient limitations in their daily activities frequently occurs due to the strong tensile and bending forces acting on the patella [13, 15]. To the best of the authors’ knowledge, there is currently no score available to objectively assess patellar healing radiologically. In contrast, for tibia and femur fractures, an established scoring system already exists to evaluate bone healing. The ‘Radiographic Union Score for Tibia (RUST)’ is a validated scoring system used to monitor the healing progress of tibial and femoral fractures using biplanar radiographs [6, 18, 20, 21]. In this scoring system, both the anterior-posterior and lateral radiographic projections of the corresponding extremity are used to provide information on the healing progress of the fracture.

The aim of this study is to validate the RUST for surgically treated patellar fractures in order to establish an objective and standardized score to evaluate bone healing. The study also aimed to assess intra- and interobserver reliability to confirm the robustness of the score.

## Materials and methods

A retrospective analysis of radiological follow-up examinations was conducted on patients who underwent surgical treatment of patellar fractures between January 1, 2013, and June 30, 2023. The study was approved by the local ethics commission (File number 23-3494-101). Thirty patients were randomly selected by sorting by case number, encompassing a total of 105 postoperative X-rays representing all stages of healing. Initially, all radiological follow-up examinations of the patients were randomized, pseudonymized, and stored on the hospital server. The modified RUST was applied to all available X-rays by three independent trauma surgeons from a Level-1 trauma center using a standardized scoring form.

**Fig. 1:**
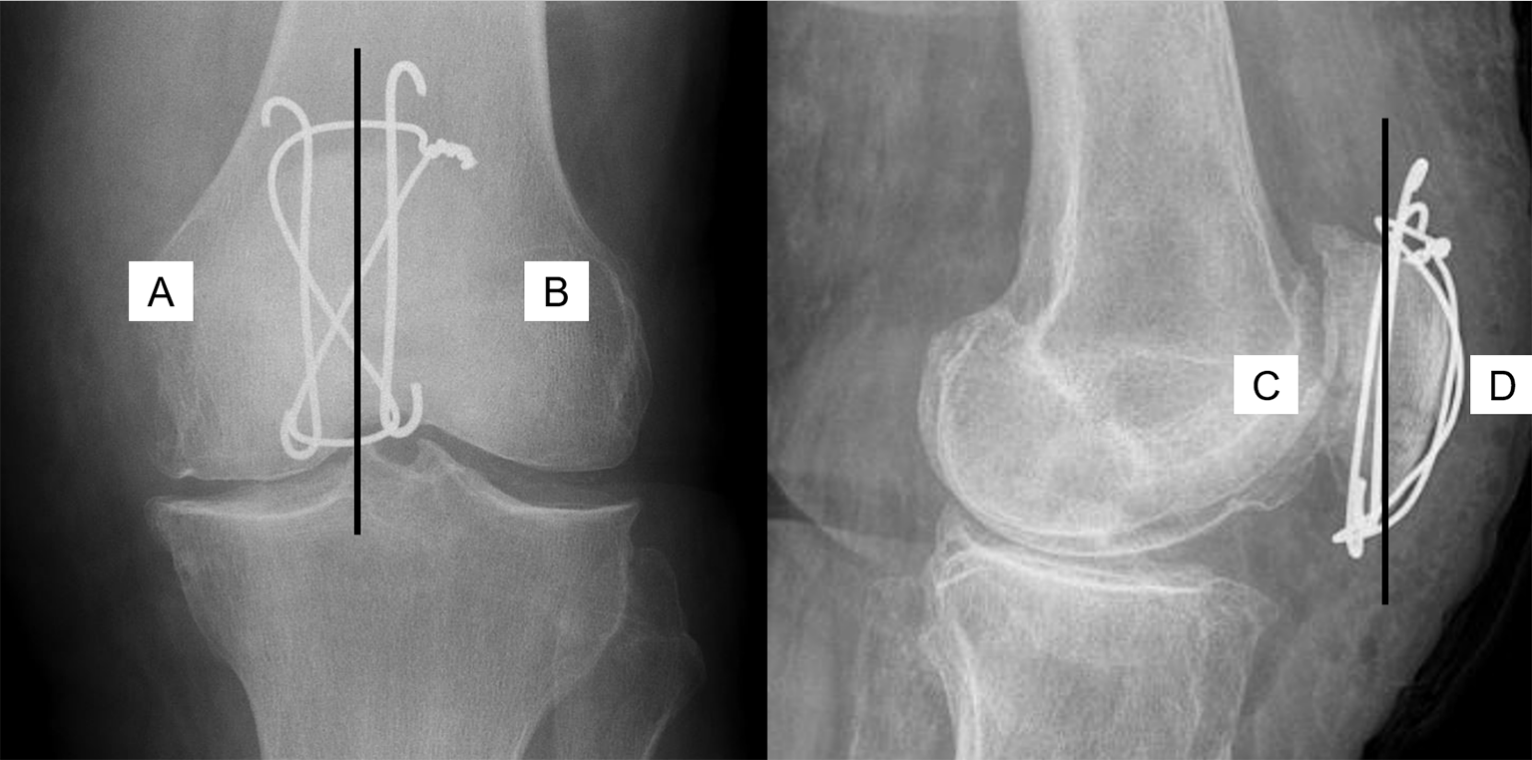
Application of the modified “Radiographic Union Score for Tibia” (RUST). **A**, **B**, **C** and **D** are to represent the individual score units

The modified RUST for the patella was applied as shown in Fig. 1. In the anterior-posterior projection, the patella was divided into medial and lateral halves. The continuity of the medial and lateral cortices was assessed. For the lateral projection, the continuity of the anterior and posterior borders of the patella was assessed in the same manner. Each border received one point if no cortical continuity was visible, two points if partial cortical bridging was present but a fracture line was still visible, and three points if complete cortical bridging was observed. The scoring system was adapted to assess direct bone healing, characterized by progressive narrowing of the fracture gap and reestablishment of cortical continuity, rather than callus formation [2]. The individual cortical scores were summed, with a total score of 4 indicating a minimum score suggesting that the fracture was not healed, and 12 indicating a maximum score suggesting complete healing. Two measurements were conducted per author with a minimum interval of two weeks, and the authors evaluated the images independently.

### Statistical analysis

The study employed the intraclass correlation coefficient (ICC) to evaluate both interobserver and intraobserver reliability. To ensure robust results, the study adhered to recommended sample size criteria by Koo et al. [12]. Interpretation of the ICC values followed guidelines akin to those used for interpreting kappa values in categorical data analysis, as proposed by Koo et al. [12]. According to these guidelines, ICC values falling within specific ranges denote varying levels of agreement: kappa < 0.5 representing “poor agreement”, 0.5–0.75 “moderate agreement”, 0.75 to 0.9 “good agreement” and > 0.9 “excellent agreement.” The ICC calculations were performed using IBM^®^ SPSS^®^ Statistics for Windows, Version 28.0, employing a two-way mixed model with absolute agreement.

## Results

**Fig. 2:**
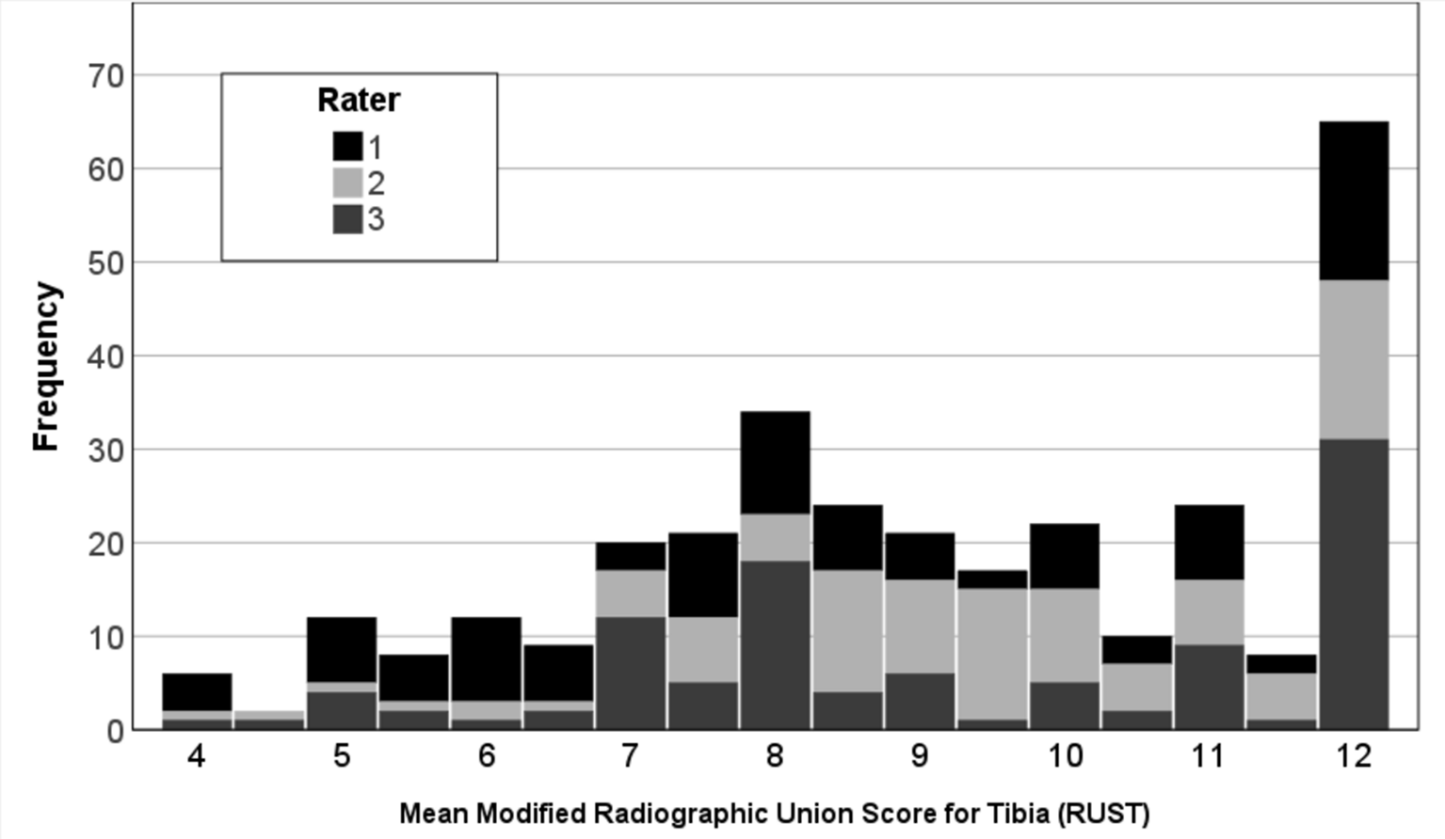
Frequency of mean modified “Radiographic Union Score for Tibia” values among 106 radiographs of osteosynthetically treated patella fractures given by three raters

Of all the X-rays reviewed, 72 depicted tension band wiring osteosynthesis, 5 depicted screw osteosynthesis, 3 depicted equatorial cerclages, and 25 depicted a combination of osteosynthesis techniques. For the 105 postoperative X-rays rated by three raters, the mean modified RUST was 9.1 ± 2.2 points, with scores ranging from 4 to 12 points (Fig. 2). The intraobserver agreement demonstrated good results for two doctors involved in the study, with ICC -values of 0.76 (0.65–0.84) and 0.80 (0.71–0.87) respectively (Table 1). Furthermore, an excellent result could be shown for one trauma surgeon with an ICC- value of 0.98 (0.98–0.99). Interobserver reliability indicated good agreement between the raters with an ICC value of 0.88 (0.81–0.92).

**Table 1:** Intra- and interobserver reliability of the modified “Radiographic Union score for Tibia” (RUST)

Reviewer	Intraobserver ICC (95% CI)	Interpretation (Koo et al.)	Interobserver ICC (95% CI)	Interpretation (Koo et al.)
1	0.76 (0.65–0.84)	Good agreement	0.88 (0.81–0.92)	Good agreement
2	0.80 (0.71–0.87)	Good agreement		
3	0.98 (0.98–0.99)	Excellent agreement		

## Discussion

Our study validated the “Radiographic Union Score for Tibia (RUST)” for assessment of bone healing in patella fractures treated with osteosynthesis. We observed good intraobserver agreement among two physicians and excellent agreement for one surgeon. Overall, interobserver reliability demonstrated good agreement.

These findings align with those of previous studies conducted on radiographs of tibial and femoral fractures. Whelan et al., who developed the score, validated it using 45 sets of radiographs of tibial fractures reviewed by seven individuals, demonstrating good overall interobserver agreement (ICC, 0.86; 95% CI, 0.79–0.91) and intraobserver reliability (ICC, 0.88; 95% CI, 0.80–0.96) [21]. Similarly, Panchoo et al. validated the RUST using 60 sets of radiographs reviewed by three individuals for adult diaphyseal femoral fractures treated with intramedullary nailing, reporting comparable results for inter- and intraobserver reliability [18]. Their interobserver agreement had an ICC of 0.87 (95% CI, 0.81–0.92), and the intraobserver agreement was 0.91 (95% CI, 0.88–0.94).

The results of our study are relevant because there is still no score available to monitor the extent of healing of the patella via conventional radiographs. Despite clear indications for surgical patella fracture treatment, it remains unclear at what time and under what circumstances the removal of the osteosynthesis material should occur [8]. A standardized scoring system could enhance comparability across clinical studies, improve the early diagnosis of delayed healing, and allow timely adjustments to therapy.

Regarding the assessment of bone healing, it is worth discussing that computed tomography (CT) is widely recognized as the superior tool for evaluating fracture healing [4, 5, 7]. However, not all clinics and outpatient centers worldwide have access to CT scanners, particularly in developing countries. With the increasing trend towards outpatient care, it is advisable to estimate and objectify healing using conventional X-ray imaging. Additionally, frequent CT scans are significantly more expensive compared to conventional X-ray diagnostics [1].

Radiation protection is also a concern that cannot be ignored, especially in young patients. Diagnostic imaging using conventional X-rays is far superior to CT in terms of radiation exposure. Koivisto et al. demonstrated that a CT scan of the knee yields effective doses ranging from 12.6 to 48 µSv, depending on the system employed, whereas plain radiographs of the knee produce only 1.8 µSv for lateral and 1.2 µSv for anterior-posterior projections [11].

Furthermore, the comparability between X-rays and CT scans poses challenges. Since CT scans should not be performed at every follow-up due to concerns about cumulative radiation exposure, X-rays offer a more practical alternative. Radiographs can be more easily compared with one another over time, theoretically allowing for better monitoring of the healing progress.

Concerning the validity of our research, the size of our cohort strengthens its robustness. Our study included 105 sets of radiographs, which is larger than those used in previously mentioned validation studies, further enhancing the reliability of our findings [3, 14, 16, 19].

### Limitations

The study design precludes making definitive statements regarding the accuracy of the score in assessing actual bone healing, as this would necessitate a comparative study with computed tomography (CT) scans, which remains the gold standard for precise evaluation of cortical alignment and continuity. The modified RUST for patellar fractures also has its limitations.

Three individuals rated the radiographs, adhering to the recommendations given by Koo et al. [12]. However, including more physicians in the study could have demonstrated the inter-rater reliability even more precisely.

Our study did not include patients who underwent plate osteosynthesis. The presence of the plate can obstruct the anteroposterior (a.p.) view of the fracture line, potentially diminishing the accuracy of the modified RUST score for this type of osteosynthesis. Furthermore, overlap from the distal femur in the a.p. view may obscure parts of the patella. However, the medial and lateral cortical edges remain visible.

There is no subchondral cortical bone, and therefore it is difficult to determine the bone bridge on the joint-facing side of the patella. While radiographs provide a practical and widely available tool, this limitation highlights the potential value of CT scans as a complementary imaging modality for cases requiring more detailed assessment.

## Conclusions

This study validated the “Radiographic Union Score for Tibia (RUST)” for evaluating bone healing in patellar fractures treated with osteosynthesis, demonstrating good intra- and interobserver reliability. The modified RUST can provide a standardised method for assessing healing in patellar fractures, benefiting both clinical practice and clinical trials.

## Data Availability

Data is available from the corresponding author upon reasonable request.
